# Parallel processing of distinct facial signals for the rapid evaluation of social agents

**DOI:** 10.1016/j.isci.2026.114978

**Published:** 2026-02-11

**Authors:** Zihe Wei, Amanda K. Robinson, Alan J. Pegna, Jessica Taubert

**Affiliations:** 1 School of Psychology, The University of Queensland, St Lucia, QLD, Australia

**Keywords:** social sciences, psychology

## Abstract

The distributed model of primate face perception proposes that distinct facial signals, such as emotional valence and sex, are processed by separate neural mechanisms. A key prediction is that cues about a face’s emotion and sex are extracted at different processing stages. To test this, we decoded time-resolved patterns of brain activity evoked by a large set of unfamiliar, naturalistic faces. Behavioral ratings were first collected to characterize the perceived emotional valence and sex of 900 faces. Electrophysiological recordings were then obtained while 40 participants passively viewed all face stimuli. The brain reliably distinguished both emotional valence and perceived sex from a brief glance, with decoding emerging in under 95 ms. Crucially, emotional valence showed earlier peak decoding than perceived sex, consistent with time-resolved representational similarity analyses. These findings indicate separable processing pipelines for changeable and stable facial signals, providing empirical support for the distributed model of face perception.

## Highlights

• The human brain extracts facial attributes from the faces of strangers in under 95 ms
• Differences in valence are represented earlier in time than differences in perceived sex
• Perceived sex relies on different cues in human faces versus non-human faces
• Different facial signals are processed by separate neural mechanisms

## Graphical abstract

**Figure:**
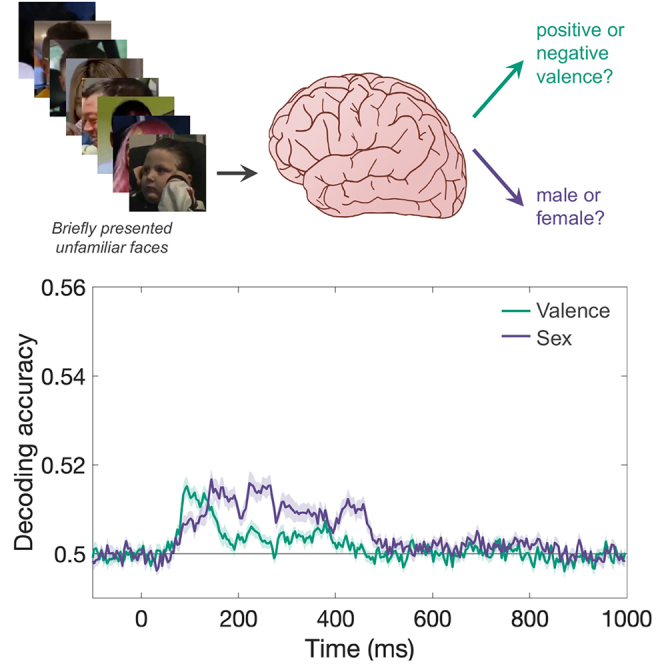

## Introduction

Being able to quickly recognize the information conveyed by a face is a uniquely important skill, and even when faces are unfamiliar, emotional valence and sex can be easily recognized.^1^^,^^2^^,^^3^ Importantly, as a changeable attribute that fluctuates from one moment to the next, emotional valence is thought to be processed separately from other more stable attributes, such as perceived sex.^4^^,^^5^^,^^6^^,^^7^ Further, emotion signals are thought to be extracted ahead of others because a person’s perceived mood directly informs decisions about approachability and social threat.^8^^,^^9^^,^^10^ However, at present, there is a dearth of brain-based evidence to support the claim that the signals underlying emotional valence judgements are extracted from the faces of strangers at an earlier processing stage than the signals underlying perceived sex judgements. In this study, our goal was to characterize the temporal dynamics underlying the perception of distinct, socially relevant, facial signals by using multivariate methods.

**Figure 1:**
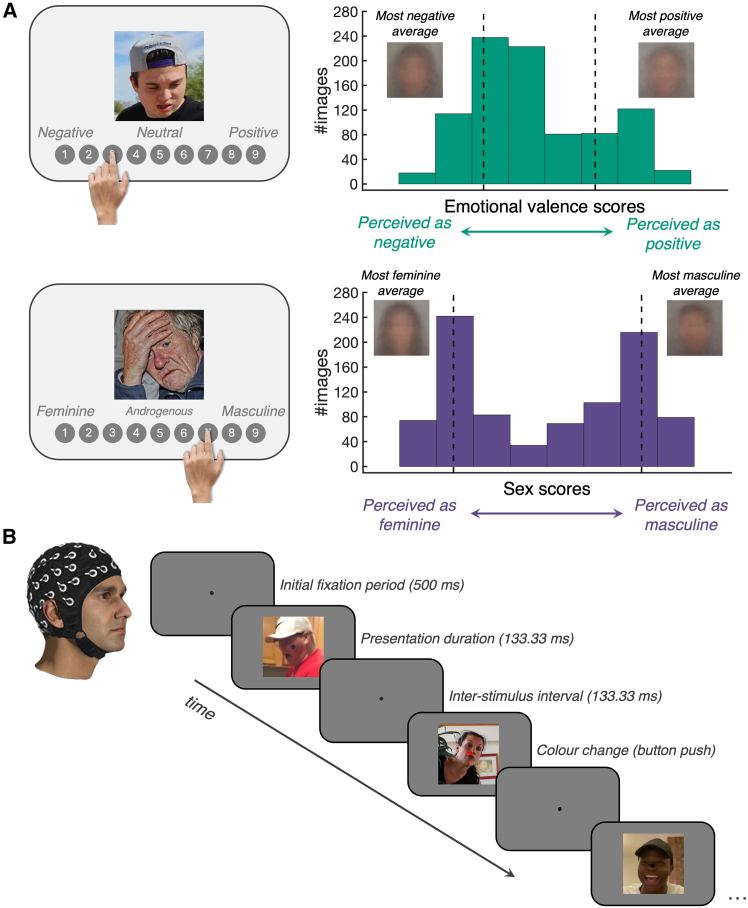
Behavioral data and experimental protocol for the EEG experiment (A) Behavioral ratings were collected for all 900 faces. On a 9-point scale, 60 people were instructed to rate “how positive or negative is this facial expression?” Similarly, on a 9-point scale, 72 people were instructed to rate “how masculine does this face appear?” Left, an illustrative example of each task. Right, histograms visualizing the distribution of average scores. Green, average scores for the perceived emotional valence. Dashed vertical lines flag the lower and upper cut-offs for images used in the decoding pipeline. The average of the images with the lowest (i.e., the 200 most negative faces) and the highest (i.e., the 200 most positive faces) valence scores is also provided. Purple, average scores for perceived sex. Dashed vertical lines flag the lower and upper cut-offs for images used in the decoding pipeline. The average of the images with the lowest (i.e., the 200 most feminine faces) and the highest (i.e., the 200 most masculine faces) sex scores is also provided. (B) Schematic of the timing parameters used in the EEG experiment. Every sequence began with a 500 ms fixation period. Thereafter, the participants were asked to fixate on a small central fixation dot and report when its color briefly changed to red.

We began by collecting behavioral ratings to classify the perceived emotional valence and sex of 900 faces taken from the Wild Faces Database^1^ (Figure 1A). The Wild Faces Database is a diverse set of 1,000 faces signaling a range of emotions,^9^ designed to test the recognition of facial signals under naturalistic levels of degradation.^11^ The set includes some non-human faces; for example it includes a number of animal faces, artwork depicting human faces, and illusory faces (i.e., face pareidolia, or the misperception of a face on an otherwise inanimate object^12^). We did not remove illusory faces from the current experiments because recent studies have shown that illusory faces carry emotional valence and perceived sex cues that can be easily recognized by human participants.^2^^,^^13^^,^^14^ Further, illusory faces have been found to elicit the same neural correlates as human faces.^15^^,^^16^^,^^17^^,^^18^^,^^19^^,^^20^ Similarly, we did not remove the artwork because they are purposely made to represent human faces. However, we did remove the animal faces because there is evidence that expertise is required to recognize facial attributes, including emotional valence.^21^^,^^22^^,^^23^^,^^24^

To determine whether there was any evidence that perceived emotion depended on perceived sex, we correlated the average scores for emotional valence (*n* = 60) with the average scores for perceived sex (*n* = 72) across the 900 faces. This yielded a reliable negative correlation, indicating that the more masculine a face appeared to be, the more negative its emotion was rated (*Spearman*′*sρ* = −0.07,*p* = 0.02,*two*-*tailed*). This observation is consistent with previous reports of a bias toward perceiving male facial expressions as more negative than female facial expressions (and vice versa)^14^^,^^25^^,^^26^ and suggests that, when we are looking at the faces of strangers, the signals for perceived emotional valence and sex could be inextricably linked.

Next, we recruited another group of participants (*n* = 40) and measured neural responses to the same 900 face stimuli by using electroencephalography (EEG). The participants sat in a dimly lit room and viewed all 900 faces, 12 times, in a brief presentation paradigm.^27^^,^^28^^,^^29^^,^^30^^,^^31^^,^^32^ In each trial, a face was presented at the center of a screen for 133.33 ms (Figure 1B). There were 150 trials in each sequence and 72 sequences in total. Thus, each subject completed 10,800 trials in total. The participants were instructed to fixate on a small black fixation dot, presented at the center of the screen, and to press the space bar whenever they saw the fixation dot turn red. These color changes occurred three to six times in each sequence. At the end of each trial sequence, the participants were encouraged to take a break. The experiment lasted approximately 1 h.

## Results

### Emotion is extracted from unfamiliar faces at an earlier processing stage than perceived sex

**Figure 2:**
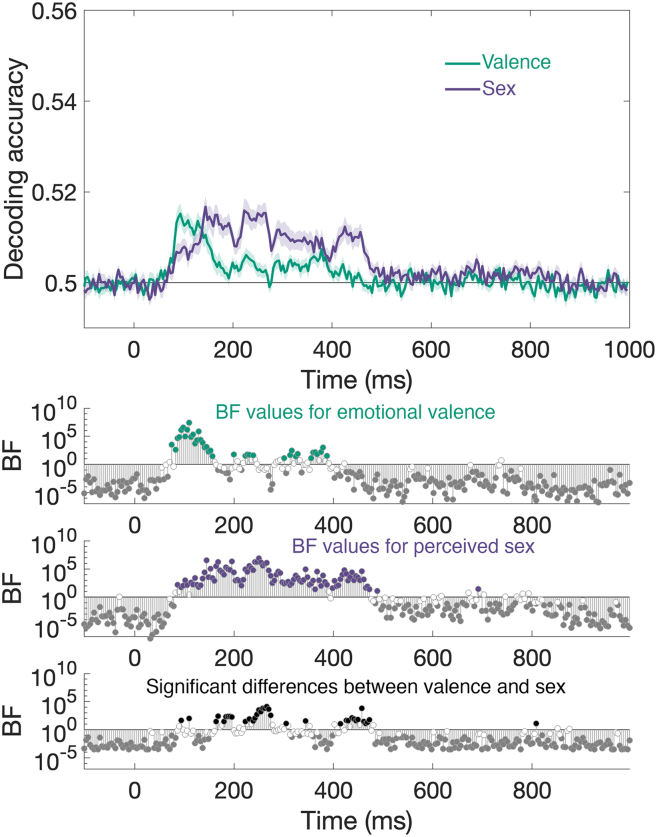
Emotional signals (negative vs. positive) are processed separately from perceived sex signals (feminine vs. masculine) Top, decoding accuracy is plotted as a function of time from stimulus onset for emotional valence (green) and perceived sex (purple). The black horizontal line marks chance performance. Bottom, three Bayes factor (BF) plots flagging when accuracy was above chance for valence (green) and sex (purple) separately. In the third plot, at the bottom, the BFs are provided for the difference between valence and sex in decoding accuracy.

We used multivariate pattern analysis (MVPA) to extract temporal information about the processing of naturalistic, unfamiliar faces from the EEG data. We took the EEG response to the 200 most negative faces and the 200 most positive faces (Figure 1A) and used time-resolved MVPA^30^^,^^31^^,^^32^^,^^33^ to determine when the difference between negative and positive faces could be reliably decoded from brain activity. Decoding accuracy as a function of time from stimulus onset is plotted in Figure 2.^15^^,^^20^^,^^27^^,^^28^^,^^30^^,^^31^^,^^32^^,^^33^^,^^34^ We then performed the same analysis for perceived sex by first selecting the 200 most feminine faces and the 200 most masculine faces (Figure 1A). Decoding accuracy as a function of time from stimulus onset is also plotted in Figure 2. For each analysis and time point, Bayes Factors (BFs) were calculated to assess the evidence that decoding accuracy was reliably above chance (i.e., BFs > 10 indicate strong evidence for the alternative hypothesis; see STAR Methods for more details). Decoding accuracy for emotional valence (i.e., distinguishing between negative and positive faces) reliably exceeded chance levels, starting at approximately 82.03 ms (95% CI [70.31, 85.94]) after stimulus onset and peaking at 93.75 ms (95% CI [89.84, 132.81], peak accuracy = 51.52%; Figure 2). In contrast, decoding accuracy for perceived sex (i.e., distinguishing between feminine and masculine faces) emerged later, becoming reliably above chance at 93.75 ms (95% CI [82.03, 132.81]) and peaking at 144.53 ms (95% CI [144.53, 265.62], peak accuracy = 51.67%; Figure 2).

Critically, BF analyses confirmed that the decoding accuracy for emotional valence was reliably greater than that for perceived sex in the early time window (93.75 and 109.38 ms, respectively), whereas the opposite pattern emerged across multiple later time windows spanning from approximately 164.06 to 473.44 ms, with decoding for perceived sex surpassing decoding for perceived valence. This pattern indicates that emotional valence cues are extracted earlier than perceived sex cues, consistent with prior work suggesting that emotional signals are prioritized due to their functional significance for social evaluation and threat detection.^9^^,^^10^^,^^25^^,^^35^

To examine the extent to which differences in low-level properties explain the different time courses reported in Figure 2, we measured average luminance (i.e., average image pixel intensity), contrast (i.e., the standard deviation of pixel intensity in each image), and spatial frequency content (i.e., both low band and high band) in each of the images used in the decoding analysis. Spatial frequency content was quantified by first applying a discrete Fourier transform to each image, then performing a median split of the resulting frequency components. The radial spatial frequency profiles of the lower and higher bands were then averaged separately. When we compared the 200 images with the lowest masculinity scores to the 200 images with the highest masculinity scores, using four independent samples *t* tests, we found no evidence that average contrast differed between these two sets of images (*t*(398) = 1.88, *p* = 0.061). Similarly, there was no evidence that high spatial frequency content differed (*t*(398) = 0.390, *p* = 0.693). However, there was evidence that both average luminance and low spatial frequency content differed (luminance, *t*(398) = 2.22, *p* = 0.027; low spatial frequency content, *t*(398) = 2.50, *p* = 0.013). When the same analyses were performed comparing the 200 images with the lowest valence scores to the 200 images with the greatest valence scores, the same pattern of differences emerged (luminance, *t*(398) = −3.91, *p* < 0.001; contrast, *t*(398) = 0.37, *p* = 0.709; low spatial frequency content, *t*(398) = −2.47, *p* = 0.014; high spatial frequency content, *t*(398) = −1.67, *p* = 0.097). On one hand, this means that the average luminance and low spatial content could be important visual cues that contribute to the classification of emotional valence and perceived sex when looking at unfamiliar faces under naturalistic circumstances. On the other hand, and importantly, because we see the same differences among the faces used for the decoding of emotional valence and for the decoding of perceived sex, these low-level properties cannot explain the differences we found in the neural time-courses (Figure 2). Therefore, these decoding results provide neural evidence for the independent and hierarchical processing of distinct facial signals, supporting models that propose temporally offset processing streams for changeable and stable facial cues. These analyses quantify, when in time, each signal becomes decodable, not how much unique variance each explains; thus, the differences we report reflect temporal dissociation, rather than statistical independence of valence and sex representations.

### Perceived emotion can be extracted from a diverse range of face stimuli

**Figure 3:**
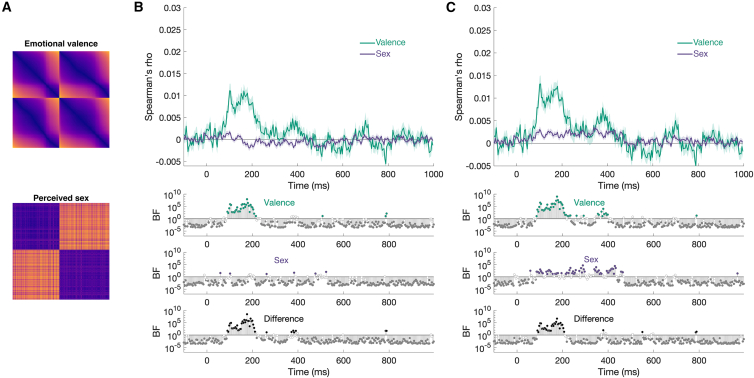
Differences in emotional valence are represented more strongly in the neural time course (A) The behavioral representational dissimilarity matrices (RDMs) for emotional valence scores (top) and perceived sex scores (bottom). (B) Top*,* Spearman’s rho, reflecting the correlation between behavioral data and neural data, plotted as a function of time from stimulus onset for emotional valence judgements (green) and perceived sex judgments (purple). The gray horizontal line marks zero correlation. Bottom, three Bayes factor (BF) plots flagging when the correlation was above zero for valence (green) and sex (purple) separately. In the third plot, at the bottom, the BFs are provided for the difference between valence and sex in correlation. Also see Figure 4. (C) Representational similarity analysis (RSA) results when the analysis was restricted to human face stimuli; conventions are the same as in (B).

To assess how neural representations of emotional valence generalize across a diverse range of naturalistic face stimuli, we conducted representational similarity analysis (RSA), using time-resolved EEG data. Neural representational dissimilarity matrices (RDMs) were computed for every time window (resolution of 3.9 ms) and compared them against the behavioral RDM derived from valence ratings through correlation (Figure 3A). The results revealed a reliable positive correlation between the brain signal and the behavioral ratings between 89.84 and 214.84 ms. In other words, the pattern of neural activity reflected that emotional valence perception started from approximately 90 ms after stimulus onset and remained reliably above chance until approximately 215 ms, suggesting a sustained neural encoding of emotional valence (Figure 3B).

**Figure 4:**
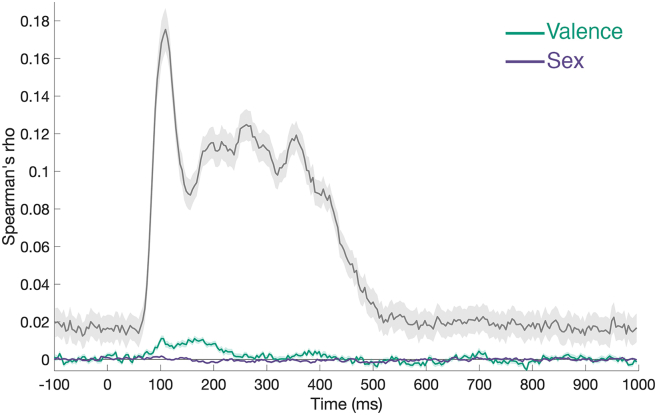
Noise ceiling estimation Data and conventions are the same as presented in Figure 3B. The gray line indicates the lower-bound estimate of the noise ceiling. Shaded regions represent ±1 SEM.

Importantly, this analysis included all 900 face stimuli and, therefore, the significant correspondence between the neural and behavioral data was observed regardless of stimulus variability, demonstrating that the brain extracts emotional valence signals consistently across different face types, including those varying in age and race, as well other social and visual characteristics. To assess the robustness of the behavioral data, we quantified the reliability of the behavioral RDM (Figure 3A, top) by using a split-half approach. The participants (*n* = 60) were randomly divided into two groups of equal size, and the correlation between their group-averaged RDMs was computed using Spearman’s rho. This process was repeated 100 times to generate a distribution of reliability estimates. When this procedure was applied to the emotional valence ratings, the average Spearman’s rho was 0.89, with all rho values greater than 0.85 (median rho = 0.89, range = [0.87, 0.9]) and all *p* values less than 0.05 confirming high internal consistency across the participants. This result demonstrates that the structure of the behavioral RDM is stable and that the participants produced highly concordant judgments of emotional valence. In sum, these findings suggest that the neural mechanisms responsible for the recognition of emotional valence are robust and generalizable, even among faces that are unfamiliar and briefly shown, supporting the idea that emotional valence is a fundamental and prioritized dimension of face perception.

In contrast, perceived sex showed weaker and less consistent correlations between neural and behavioral RDMs. While a small, transient effects were observed at a number of time points, these time points were not contiguous and, therefore, are not considered statistically reliable. To evaluate whether this reflected increased variability in the behavioral data, we repeated the same split-half procedure for the perceived sex ratings (*n* = 72). This analysis revealed that the perceived sex ratings were also highly reliable, with a mean Spearman’s rho of 0.92, all rho values greater than 0.9 (median rho = 0.92, range = [0.91, 0.94]), and all *p* values less than 0.05. This confirms the strong agreement among the participants who were tasked with judging the perceived sex of the faces. Together, these results indicate that the behavioral data for both attributes are internally consistent and that the differences observed in neural decoding likely reflect genuine differences in the stability of the underlying neural representations, rather than noise or variability, in the behavioral ratings.

### Signals underwriting perceived sex are more consistent among human faces

Initially, when the RSA was performed across all 900 face stimuli, including non-human faces (e.g., pareidolia images and paintings), the correlation between neural and behavioral RDMs for perceived sex was weak and not statistically reliable (see Figure 3B). This suggests that the visual cues driving the classification of perceived sex vary substantially across diverse face types, leaving the encoding of perceived sex less consistent when considering a broad set of stimuli. Thus, in Figure 3C, the RSA was restricted strictly to human faces. This approach uncovered a reliable positive correlation between the neural and behavioral data for both emotional valence and perceived sex (Figure 3C). For sex, this effect became reliable at 85.94 ms after stimulus onset and largely persisted until 464.84 ms, indicating that the neural representation of perceived sex is more stable and reliable when analyzing human faces exclusively. These results suggest that the brain encodes perceived sex in a more structured manner when restricted to processing human faces. This suggests that naturalistic images of human faces contain reliable sexually dimorphic features, for example, possibly facial shape, hair length, and secondary sexual characteristics.^36^ In contrast, the cues that support the judgment of perceived sex when looking at non-human faces, such as the faces of toys and illusory faces in objects, might be less reliable. This highlights the potential importance of species-specific priors in the perception of sex.

To confirm that the information available in the behavioral RDM was reliable across participants once the non-human face stimuli were removed, we re-ran the split-half reliability analysis described above, using only behavioral data associated with the human face stimuli. For emotional valence and perceived sex, the distribution of rho values was similar (emotional valence, mean rho = 0.89, median rho = 0.89, range = [0.87, 0.91]; perceived sex, mean rho = 0.91, median rho = 0.92, range = [0.9, 0.93]), indicating that the scores assigned to human faces in both behavioral tasks were reliable across the participants.

## Discussion

Our goal was to understand how different socially relevant signals are extracted from the faces of people that have never been seen before. This is an incredible computational feat considering the sheer number of strangers we encounter on a daily basis, all of which are automatically evaluated for potential social threat to facilitate vigilance any necessary preparatory behaviors.^37^^,^^38^^,^^39^ While behavioral evidence has suggested that cues underlying emotional valence and perceived sex judgements are not only important to recognize but are also contingent on each other, with angry people appearing more masculine, and vice-versa.^14^^,^^25^^,^^26^ Neural models have emphasized the independence of the mechanisms responsible for processing cues such as emotion and sex. Here, we show that asking participants to recognize emotion cues in a large set of naturalistic faces evokes a dynamic neural signature that is entirely distinct from when asking participants to recognize sex cues in the same faces. These findings provide new insights into how the human brain extracts social knowledge from faces for the purpose of rapidly evaluating unfamiliar people.

The distributed processing model of face perception posits that changeable facial signals, like emotional expression, gaze direction, and mouth shape, must be processed by distinct mechanisms from more stable signals, like sex, race, and identity, due to their opposing computational demands.^4^^,^^6^^,^^40^^,^^41^^,^^42^^,^^43^^,^^44^^,^^45^ For example, to accurately recognize an emotional expression, a mechanism has to ignore all the cues and features in a face that remain constant over time and, instead, monitor dynamic cues that are constantly changing. However, to accurately recognize a face’s identity, a mechanism has to ignore all the dynamic cues and distil the stable cues and features that transcend circumstance.^40^^,^^42^^,^^46^ While patient-based evidence has provided some support for the distributed face-processing model,^47^ it continues to lack strong empirical support.^7^^,^^48^ This could be, at least in part, because tests of the distributed face processing model have often compared the recognition of facial expressions to the recognition of facial identities, and identity judgements require the face stimuli to be familiar to the participants. Recent studies have shown that familiar faces drive a vastly different response from the entire human brain compared with unfamiliar faces^49^^,^^50^^,^^51^^,^^52^^,^^53^ and, thus, a reliance on identity judgments and familiar face stimuli, exclusively, might be constraining or obscuring scientific progress.

Our capacity for extracting social cues from the faces of strangers has been best demonstrated in studies of social evaluation; we are equipped to quickly determine how pleasant and approachability someone appears based on facial signals.^35^^,^^54^^,^^55^^,^^56^ Importantly, these impressions have been shown to impact real-world outcomes, from electoral success^57^ to financial gain^58^ and judicial decisions.^59^ Yet, the processes that underwrite the perception and recognition of social signals in unfamiliar faces remain understudied. Here, we addressed this knowledge gap by looking at the link between behavioral and neural responses to a large set of naturalistic faces.

Figure 2 shows that the emotional valence and sex labels that were given to 900 photographs of different faces could be successfully decoded from brain activity. More importantly, the onset and the peak decoding performance occurred at different times for the two facial attributes with the pattern, suggesting that faces with positive and negative emotions are distinguished before faces with feminine and masculine features are distinguished (Figure 2). This is consistent with a large body of evidence suggesting that the emotion conveyed by different facial behaviors and expressions is prioritized over other facial signals and consequently recognized much more rapidly, perhaps even prior to awareness.^8^^,^^10^^,^^38^^,^^60^ Although there is some debate about the cortical loci that might be responsible for expression recognition,^7^^,^^61^ many researchers have argued that subcortical structures, such as the amygdala, are tuned to differences in emotional valence^7^^,^^8^^,^^62^^,^^63^^,^^64^^,^^65^^,^^66^^,^^67^^,^^68^^,^^69^^,^^70^ and are, therefore, likely to also play a role in the recognition of facial expressions. Collectively, all of our findings in Figures 2 and 3 demonstrate that emotional valence, whether the expression is positive or negative, can be reliably recognized from even a brief glance at a face.

It is important to note that low-level visual properties such as luminance, contrast, and spatial frequency likely contribute to the perception of both emotional valence and perceived sex, as higher-level representations necessarily emerge from analyses of these lower-level inputs. However, our analyses revealed that the magnitude of these low-level differences was comparable across the image sets used for decoding emotional valence and perceived sex. Thus, while such properties may contribute to both forms of classification, they cannot explain the distinct temporal profiles observed in the neural data. These differences in temporal dynamics, therefore, indicate that the classification of valence and perceived sex, even if informed by similar sources of visual information, occurs at separate computational stages. More broadly, by using naturalistic face stimuli that preserve the typical variability in low-level features found in real-world encounters, our approach captures the ecological complexity of social vision and provides insight into how the human brain encodes multiple facial signals under natural viewing conditions.

In contrast, little is known about the neural mechanisms underlying the recognition of sex in faces. Indeed, this is often studied in the context of familiar faces, where it has been shown that cues underlying the perception of sex are extracted exceedingly quickly (∼70 ms after stimulus onset).^71^ In Figure 2, we show that for faces that are easily classified as being either feminine or masculine, perceived sex is encoded quickly (∼94 ms), albeit later in time than that previously reported for familiar faces^12^ (Figure 2). Interestingly, unlike for emotional valence, when the variation across all 900 images was included to look at the relationship between behavioral ratings and brain activity (Figure 3A), we found little evidence that perceived sex was being reliably encoded by the human brain. This result suggests that the visual cues distinguishing feminine and masculine faces vary considerably across pairs of faces. This was somewhat confirmed because when we removed the non-face stimuli, which included paintings, objects with illusory faces, and toys, we recovered evidence that perceived sex was being reliably encoded for human face stimuli (Figure 3B). What this might suggest is that while emotional cues could be shared across human and non-human faces, this is not the case for the cues underlying sex judgements. It follows that different visual cues might be used to distinguish masculine and feminine faces in biological agents compared with non-biological agents. While this study is not designed to determine what those visual cues might be, as there was only a small number of non-face items included in the design (i.e., 60 images of non-faces compared to 840 images of human faces), it seems possible from the average faces in Figure 1B that at least two of the cues often relied upon to discern the sex of an unfamiliar face is the length of hair on the head, and/or the presence of facial hair on the chin.^36^ Given the evidence that inanimate objects that merely look like faces evoke the same neural signature as human faces,^15^^,^^16^^,^^17^^,^^18^^,^^19^^,^^72^^,^^73^^,^^74^^,^^75^ further research is warranted in the future.

Another important consideration when comparing the emotional valence and perceived sex ratings data is the potential for differential ambiguity. For example, if the participants assigned to the emotional valence task understood the question and the task at hand better than those assigned to the perceived sex task, then this could explain the earlier and stronger correlations with the neural data associated with emotional valence. We noted that, in both cases, the participants in the behavioral rating experiments were left to define “positive and negative expressions” as well as “masculine and feminine faces” based on their own comprehension and experience, as these were online tasks with no instructor available to provide any further clarification. Further, using split-half reliability analyses, we found that both the emotional valence ratings and the perceived sex ratings were reliable across the participants. Because there is no clear evidence that one task was better understood than the other, we argue it is more likely that the differences we report between emotional valence and perceived sex signals reflect the differences in neural processing. Future research could explore whether emotional valence and perceived sex tasks could be equated for task difficulty.

These data reveal that the temporal signature associated with the encoding of emotional valence is profoundly different from the temporal signature associated with the encoding of perceived sex (Figure 2). Importantly, these differences indicate that emotional valence and perceived sex are processed at distinct computational stages in time but do not imply that the underlying representations are statistically independent. Initially, decoding performance is stronger for emotional valence than for perceived sex, but this reverses by ∼164 ms after stimulus onset, after which decoding performance is stronger for perceived sex than for emotional valence. This suggests that these different facial signals are processed at different computational stages, and possibly by separable mechanisms.^1^^,^^2^^,^^3^^,^^4^^,^^5^^,^^6^^,^^7^^,^^8^ Given these results, it is reasonable to assume that other changeable facial attributes, such as gaze direction/head orientation,^76^^,^^77^ arousal,^78^ or health^79^ would also evoke temporal signatures that are distinct from those evoked by other stable facial attributes, such as identity^5^ or perceived race.^80^ These would need to be tested in future experiments. Additionally, it would be of interest to understand how an explicit task would change the neural encoding of facial attributes. Although previous research has shown that the inclusion or manipulation of a participant’s task does not alter the early encoding of visual stimuli,^27^^,^^81^ it remains possible that asking participants to respond to specific facial attributes would change the neural activity associated with later cognitive processes. In sum, our current findings show that the extraction of different facial attributes is hierarchical, with the extraction of coarse emotional cues occurring before the diagnostic cues associated with perceived sex can be evaluated. These findings inform broader questions about how multiple, complex, socially relevant signals can be extracted from face stimuli to effectively inform active vision and behavioral goals.

### Limitations of the study

This study focused on the temporal dynamics of neural responses to unfamiliar faces during passive viewing. As such, the results do not address how explicit task demands, attention, or goal-directed behavior may modulate the timing or strength of facial attribute encoding. Additionally, while the stimulus set was intentionally naturalistic and heterogeneous, this variability limits the precise identification of specific visual cues driving emotional valence and perceived sex judgements, particularly for non-human or illusory faces. Although control analyses showed that low-level image properties could not account for the observed temporal dissociations, future work using computational feature modeling could further clarify the cues supporting each judgment. Finally, the use of EEG provides excellent temporal resolution but limited spatial specificity; complementary neuroimaging or intracranial recordings would be required to localize neural sources underlying the observed effects.

## Resource availability

### Lead contact

Further information and requests for resources should be directed to and will be fulfilled by the lead contact, Jessica Taubert (j.taubert@uq.edu.au).

### Materials availability

This study did not generate reagents.

### Data and code availability

All data have been deposited at the UQ espace and are publicly available as of the date of publication. Accession number is listed in the key resources table.

All statistical analyses were performed using MATLAB. All scripts are available online via UQ espace: https://doi.org/10.48610/b58aac1. Any additional information needed to reanalyze the data reported in the paper will be provided by the lead contact upon request.

## Acknowledgments

This research was supported by the 10.13039/501100000923Australian Research Council (FT200100843 to J.T. and DE200101159 to A.K.R.).

## Author contributions

Z.W. designed the study, collected data, performed the final analyses, and wrote the article; A.K.R. designed the study, supervised the final analyses, and wrote the article; A.J.P. designed the study and supplied the resources; J.T. designed the study, acquired funding, supervised the final analyses, and wrote the article. All authors have approved the final version of the article.

## Declaration of interests

The authors declare no competing interests.

## STAR★Methods

### Key resources table

**Table:** 

REAGENT or RESOURCE	SOURCE	IDENTIFIER
**Software and algorithms**		

Qualtrics	https://www.qualtrics.com/	RRID:SCR_016728
Matlab_R2023b	https://au.mathworks.com/	RRID:SCR_001622
Psychophysics Toolbox	http://psychtoolbox.org/	RRID:SCR_002881
EEGLAB	https://sccn.ucsd.edu/eeglab/	RRID:SCR_016333
CoSMoMVPA	https://cosmomvpa.org/	RRID:SCR_014519
Psychopy2	https://www.psychopy.org/	RRID: SCR_006571

**Deposited data**		

UQ espace	https://doi.org/10.48610/b58aac1	–

### Experimental model and study participant details

We performed three experiments that investigated the temporal dynamics of brain activity when participants viewed naturalistic facial stimuli: an expression valence rating task, a perceived sex rating task, and an EEG study. All participants were recruited from the University of Queensland SONA system in return for course credits. For the emotional valence ratings, we recruited 60 participants (45 females, 15 males; mean age: 19.44, SD: 3.89). For the perceived sex ratings, we recruited 72 participants (56 females, 16 males; mean age: 19.69, SD: 3.45). For the EEG component, 44 participants were recruited. Four participants were excluded due to equipment failure, and data from the 40 remaining participants were analysed (27 females, 13 males; mean age: 21.6, SD: 5.26). Subjects reported normal or corrected-to-normal vision and no history of psychiatric or neurological disorders.^46^ We did not record participants’ ancestry, race, ethnicity, and socioeconomic status because demographic and social variables were not our focus, but we acknowledge that the absence of data on these factors may limit the generalizability of our findings. The study was approved by the Human Ethics Committee of the University of Queensland (Ethics committee approval code: 2023/HE000783). Verbal and written consent was obtained from each participant.

### Method details

#### Stimuli

Stimuli were 900 naturalistic facial images (see Figure 1 for examples). We used images of 839 human faces and 60 non-faces (e.g., pareidolia images, painting of a face, toys) from the Wild Faces Database^9^ and added another human face to the stimulus set to result in 900 images. All images were cropped to a square aspect ratio. No other filtering or editing was applied to the stimuli to provide a naturalistic demonstration of visual processing.

#### Procedure

##### Ratings tasks

Behavioural testing was conducted online via Qualtrics to obtain emotional valence and perceived sex ratings for the 900 naturalistic face stimuli. In this study, we refer to “perceived sex” rather than “sex” or “gender” because the biological sex and the sexual identity of the individuals in the stimulus set are unknown. As the classification of each image as feminine or masculine is derived solely from participant ratings, we consider perceived sex to be the most accurate description of this construct.

Each participant rated 300 of the total 900 images, selected randomly. The order of image presentation was randomised, and each image was only presented once. Each task took approximately 20 minutes to complete. For the emotional valence task, participants (*N* = 60) were asked to rate, on a scale from one to nine, “how positive or negative is this facial expression?”. The scale ranged from one being extremely negative to nine being extremely positive, and five being neutral (see Figure 1A). In a separate experiment, we obtained perceived ratings for perceived sex. For each face, participants (*N* = 72) were asked “how masculine does this face appear?” and had to rate their response on a scale ranging from one to nine, with one being very feminine to nine being very masculine, and five being androgynous (see Figure 1A). For each task, data from all participants were collated and the group mean rating was calculated for each stimulus.

##### EEG session

In a separate group of participants, we measured neural responses to the same naturalistic face stimuli using electroencephalography (EEG). The experiment was presented in Psychopy2.^82^ Participants sat in a dimly lit room approximately 57 cm away from a 1920 × 1080 pixel Asus computer monitor, and the stimuli subtended approximately 8 degrees visual angle. Stimuli were presented on a grey background with a black fixation dot (0.5 degrees visual angle) superimposing the stimuli. Images were then presented in sequences of 150 stimuli (133.33 ms image duration and 133.33 ms inter-stimulus interval), with each sequence lasting approximately 40 seconds (Figure 1B). At the start of a sequence, a fixation dot was presented for 500 ms. Every six consecutive sequences (one “block”) contained all 900 unique stimuli in random order. There were 72 sequences in total, consisting of 10,800 image trials (12 repeats of each of the 900 stimuli).

In each sequence, participants were instructed to fixate upon a black dot superimposed over each stimulus at the centre of the screen and told to respond by pressing the space bar whenever they spotted the fixation dot turn red. Fixation colour changes were randomised to occur between three and six times in each sequence. The onset and duration of the colour change corresponded with one image presentation (i.e., viewed for 133.33 ms). At the end of each sequence, the display showed the progress through the experiment, and participants were able to start the next sequence with a button press. Participants were asked to sit still and minimise eye movements during the sequences and to use the time between sequences as breaks. The experiment lasted approximately one hour.

Continuous EEG data were recorded using a 64-electrode Biosemi EEG system at a sample rate of 1024 Hz. Electrodes were placed in accordance with a 10/20 international system.^83^ An event trigger was sent over the serial port at the start of each sequence (trigger code 3), at every stimulus onset event (trigger code 1), at every target onset (trigger code 2), and at every response (trigger code 5).

Pre-processing of the EEG data was computed offline using EEGLAB.^84^ The continuous EEG data were temporarily referenced to Cz, then filtered with a high-pass filter of 0.1-Hz and a low-pass filter of 100-Hz. If a channel signal surpassed a threshold of five standard deviations from the mean probability distribution, then it was considered a noisy channel and interpolated. Subsequently, the EEG data were re-referenced to the average of all electrodes and down sampled to 256 Hz. No notch filter was applied. The data were then separated into epochs corresponding to each stimulus presentation, ranging from 533 ms pre- to 1067 ms post-stimulus onset (i.e., epoch duration equal to six image presentation cycles in the experiment). Linear detrending was applied to each epoch, then the time window was cropped to -100 ms pre- to 1000 ms post-stimulus onset. This produced 10,800 pre-processed epochs for each participant.

### Quantification and statistical analysis

#### Decoding analysis

For each subject, we used time-resolved multivariate classification (“decoding”) to discriminate between all pairs of images in the stimulus set. Then, we ran time-resolved decoding analyses of valence and sex. For all decoding analyses, analysis of EEG data was implemented in MATLAB with the CoSMoMVPA toolbox.^85^ We applied Linear Discriminant Analysis (LDA) classifiers^86^ separately for each participant and each time point in the epoch.

For the image decoding analyses, we performed classifications across all 900 stimuli, resulting in 404,550 unique image pairs. We implemented a leave-one-block-out cross-validation scheme with 12 blocks, where the classifier was trained on 11 blocks and tested on the held-out block. This process was repeated 12 times, with each block serving once as the test set. We calculated the mean of these pairwise analyses as a measure of image specificity in the neural signal (chance = 50%).

Next, we investigated whether images from different classes (i.e., masculine/feminine, positive/negative expression) evoked separable neural signals. We selected the highest and lowest 200 images based on the valence and sex ratings respectively, resulting in a total of 400 stimuli per rating experiment (see Figure 1 for high/low cut-offs). This choice of stimuli was designed to maximise the contrast between these classes. Classifiers were trained to discriminate the two classes (e.g., high versus low valence) on all but one exemplar of each class in 11 session blocks (training on 199 exemplars per class) and testing on the left-out exemplars in the remaining block. This process was iterated for 200 exemplar pair combinations and 12 blocks. Thus, successful classification would indicate that neural patterns associated with each class generalised to novel stimuli, rather than relying on individual image-specific properties.

The LDA classifiers used whole brain data from the 64 EEG sensors to estimate the probability of EEG data belonging to a certain class (e.g., negative or positive) where the higher estimate is the predicted class.^86^ This was repeated at every time point, for every participant, and averaged across subjects to generate the mean cross-validation decoding performance at each time point. Classification performance was assessed using Bayesian statistics to compare decoding accuracy to chance level (>50%) as described below. Above-chance decoding accuracy informs us that the EEG data contains information relevant to the contrast of interest.^34^^,^^87^

#### Representational similarity analysis

To complement the decoding analyses, we used representational similarity analysis (RSA)^88^^,^^89^^,^^90^^,^^91^ to assess how neural responses captured information about perceived emotional valence and perceived sex. For each EEG participant and at each timepoint, we constructed neural representational dissimilarity matrices (RDMs) by computing all pairwise dissimilarities between stimulus-evoked patterns in the EEG data. Each RDM was a 900 × 900 matrix corresponding to all pairwise comparisons among the 900 stimuli, yielding 404,550 unique dissimilarity values. Behavioural RDMs were created separately for perceived valence and perceived sex, based on the absolute difference in the mean behavioural ratings assigned to each stimulus pair. Neural RDMs were then compared to these two behavioural RDMs using Spearman rank-correlation, producing a time-resolved measure of correspondence between neural and behavioural representational geometry. The correlations computed for each EEG participant and then averaged across the group. To evaluate statistical evidence for representational correspondence, Bayes factors were estimated for each timepoint relative to a null correlation of zero. This analysis followed standard RSA practices described in prior studies.^30^^,^^32^^,^^33^^,^^92^^,^^93^

#### Noise ceiling estimation

To estimate the maximum explainable variance in the neural data, we calculated a noise ceiling that reflects the upper limit of model performance given the inherent noise and inter-participant variability in EEG responses. At each time point, each participant’s neural representational dissimilarity matrix (RDM) was rank-transformed and then Spearman-correlated with the mean rank-transformed RDM computed from all other participants (leave-one-participant-out approach). The average of these correlations across participants provided the lower-bound estimate of the noise ceiling (see Figure 4). This procedure establishes how well any model could theoretically predict the neural data, allowing the observed model correlations to be interpreted relative to the reliability of the underlying signal.^93^^,^^94^

#### Statistical inference

Bayesian statistics were used to quantify the strength of evidence for or against the presence of an effect at each time point.^95^ Analyses were conducted using the “BayesFactor” package in RStudio (version 4.4.0). For the decoding analyses, Bayes factors (BFs) compared the likelihood of the alternative hypothesis, that classification accuracy exceeded chance, against the null hypothesis of chance-level performance. To evaluate differences between conditions (e.g., emotional valence vs. perceived sex), we computed Bayes factors for contrasts of paired samples, quantifying the evidence that decoding accuracy or correlation values for one attribute exceeded those for the other. This approach directly assessed the temporal dissociation between the two facial attributes. For the RSA, BFs tested whether the Spearman correlations between neural and behavioural RDMs were reliably above or below zero.

Bayes Factors were calculated using a Jeffreys–Zellner–Siow (JZS) prior, centred on the null (chance) value^96^ with default scale factor of 0.707. For the alternative hypothesis, 50% of parameter values were expected to fall within ±0.707 standard deviations from chance.^96^^,^^97^ For decoding accuracy, the null interval was specified as a range of effect sizes between 0 to 0.5^98^ (i.e., one-sided tests to assess above-chance accuracy), and for correlations the null interval was specified as a range of effect sizes between -.5 to .5 (i.e., two-sided tests).

*BF*s quantify the relative strength of evidence supporting the alternative versus null hypotheses. A BF greater than one supports the alternative hypothesis, and a BF less than one supports the null. Although BFs represent a continuous measure and should not be strictly categorised, conventional interpretations suggest that BFs > 3 indicate moderate evidence and BFs > 10 indicate strong evidence for the alternative hypothesis. Conversely, *BFs* < 1/3 and < 1/10 indicate moderate and strong evidence in favour of the null hypothesis, respectively. In sum, for all time-resolved analyses, BFs quantify the strength of evidence that decoding accuracy (Figure 2) or neural–behavioural correlations (Figure 3) differ from chance, with values above 3 and 10 conventionally interpreted as moderate and strong evidence, respectively. Additionally, because these analyses were time-resolved, inference was based on the overall temporal pattern rather than isolated points. Single time points were not considered conclusive unless neighbouring samples showed consistent evidence in the same direction.^99^ This approach reduces false positives and aligns with current best practices for interpreting time-series decoding and RSA data.^30^^,^^31^^,^^32^^,^^33^^,^^93^ All Bayes factor analyses were therefore used to evaluate temporal dissociations in effect strength rather than partitioning unique variance components.
